# Radiation-induced thermal conductivity degradation in LiAlO_2_ and LiAl_5_O_8_ investigated by molecular dynamics

**DOI:** 10.1038/s41598-025-26441-y

**Published:** 2026-01-08

**Authors:** Ankit Roy, Andrew M. Casella, Ram Devanathan, Ayoub Soulami, David J. Senor

**Affiliations:** Pacific Northwest National Laboratory, Richland, WA 99354 USA

**Keywords:** Molecular dynamics, Thermal conductivity, Radiation damage, Displacement cascades, Damage accumulation, PDOS, LiAlO_2_, LiAl_5_O_8_, Materials science, Physics

## Abstract

Radiation damage in lithium ceramic materials can severely degrade thermal transport properties, limiting their performance in nuclear environments such as tritium-producing burnable absorber rods (TPBARs). This study compares the intrinsic thermal conductivity degradation in single crystals of LiAlO_2_ and LiAl_5_O_8_ due to radiation-induced point defects. LiAlO_2_ shows a significant drop in thermal conductivity of up to 75% under increasing defect concentration and temperature, while LiAl_5_O_8_ retains over 50% of its thermal conductivity, even at high defect levels and elevated temperatures. The greater resilience of LiAl_5_O_8_ is attributed to its structural resilience, which suppresses defect generation and preserves phonon transport. Partial phonon density of states analysis reveals that Li and Al vacancies strongly suppress vibrational modes in LiAlO_2_, while LiAl_5_O_8_ shows minimal change, supporting its superior radiation tolerance. These results suggest LiAl_5_O_8_ to be a more durable candidate for high-temperature radiation environments.

**Supplementary Information:**

The online version contains supplementary material available at 10.1038/s41598-025-26441-y.

## Introduction

Gamma-phase lithium aluminate (γ-LiAlO_2_) is a critical ceramic material used in tritium-producing burnable absorber rods (TPBARs), where it serves as a lithium host to support US defense needs^1–9^. Its desirable thermodynamic stability and low neutron-induced swelling makes it an attractive candidate for extended neutron exposure environments. γ-LiAlO_2_ is being considered for use in solid breeder blankets for fusion reactors due to its chemical stability and lithium transport properties^10^. Recent experimental work has revealed that γ-LiAlO_2_ undergoes irradiation-induced phase transformation^11^. Under 200 keV electron irradiation at room temperature, precipitation of the spinel-phase LiAl_5_O_8_ has been observed, attributed to Li and O displacements in the lattice via the reaction 5 LiAlO_2_ → LiAl_5_O_8_ + 4 Li_i_ + 2 O_i_ where suffix *i* denotes interstitials^12^. The impact of such defect generation and phase transformation on the thermal conductivity (κ) of γ-LiAlO_2_ and LiAl_5_O_8_ remains largely unknown. Limited first-principles and modeling work has been conducted to estimate thermal conductivity and phonon transport in pristine γ-LiAlO_2_^13–15^ but systematic atomistic studies under irradiation and defect conditions are absent from the literature.

By contrast, the thermal transport behavior of conventional nuclear fuels such as uranium dioxide (UO_2_)^16–22^, thorium dioxide (ThO_2_)^23–28^, and plutonium dioxide (PuO_2_)^29–32^ has been extensively investigated through both experimental and computational methods. These fluorite-structured oxides have been the subject of molecular dynamics (MD) simulations and first-principles studies examining the effects of fission products, vacancies, and irradiation-induced clusters on phonon scattering and thermal conductivity. Rahman et al.^33^ demonstrated that both isolated and clustered defects significantly degrade ThO_2_ thermal conductivity, with clustered defects having a stronger impact. Malakkal et al.^28^ combined simulations and experiments to show the impact of porosity on ThO_2_ thermal transport, while similar work in UO_2_ has led to the development of analytical models used in reactor fuel performance codes. Ma et al.^26^ showed that Pu doping reduced thermal conductivity in ThO_2_ more than U doping, while Park et al.^25^ found that vacancy defects had a more pronounced effect on conductivity than uranium substitution. Martin et al.^34^ analyzed thermal expansion and diffusion in doped thoria. Lee et al.^35^ performed MD simulations of UO_2_ with nanoscale pores and found that traditional porosity-based models often overpredict conductivity, whereas the model proposed by Alvarez et al.^36^, which incorporates phonon hydrodynamics and pore size effects, aligns more closely with MD results. These modeling efforts have advanced understanding of phonon-defect interactions in actinide oxides, but similar investigations have not been extended to lithium-based ceramics.

Here we present an atomistic investigation of thermal conductivity degradation in perfect crystals of γ-LiAlO_2_ and LiAl_5_O_8_ without grain boundaries or pores, under irradiation conditions. Our study shows how temperature, irradiation-induced defect accumulation, and intrinsic point defects impact thermal transport in these ceramics. This method covers both early-stage damage and high-defect-density regimes. By comparing the response of γ-LiAlO_2_ and LiAl_5_O_8_ under identical defect conditions, we uncover fundamental differences in their damage resistance showing the superior radiation tolerance of the spinel phase. Our findings demonstrate that γ-LiAlO_2_ suffers significantly greater thermal conductivity degradation under both irradiation and vacancy-driven disorder than LiAl_5_O_8_. These results help guide future material design for TPBAR applications.

## Methods

### Simulation setup and equilibration

All MD simulations were carried out using the LAMMPS (Large-scale Atomic/Molecular Massively Parallel Simulator) package^37^. Supercells of γ-LiAlO_2_ and LiAl_5_O_8_ were constructed with periodic boundary conditions applied in all three dimensions to approximate bulk behavior. For benchmarking initial thermal conductivities, simulation cells ranging from lengths 100 Å to 900 Å were used to get the $\:\kappa\:-L$ relationship as used in prior work^27,38,39^. Initial geometries were relaxed through conjugate-gradient energy minimization to eliminate residual stresses. Systems were then equilibrated using the isothermal-isobaric (NPT) ensemble at target temperatures ranging from 300 K to 900 K. Temperature and pressure were controlled using Nose–Hoover thermostats and barostats, and equilibration was done for 20 ps.

### Benchmarking interatomic potentials and thermal conductivity methods

**Fig. 1:**
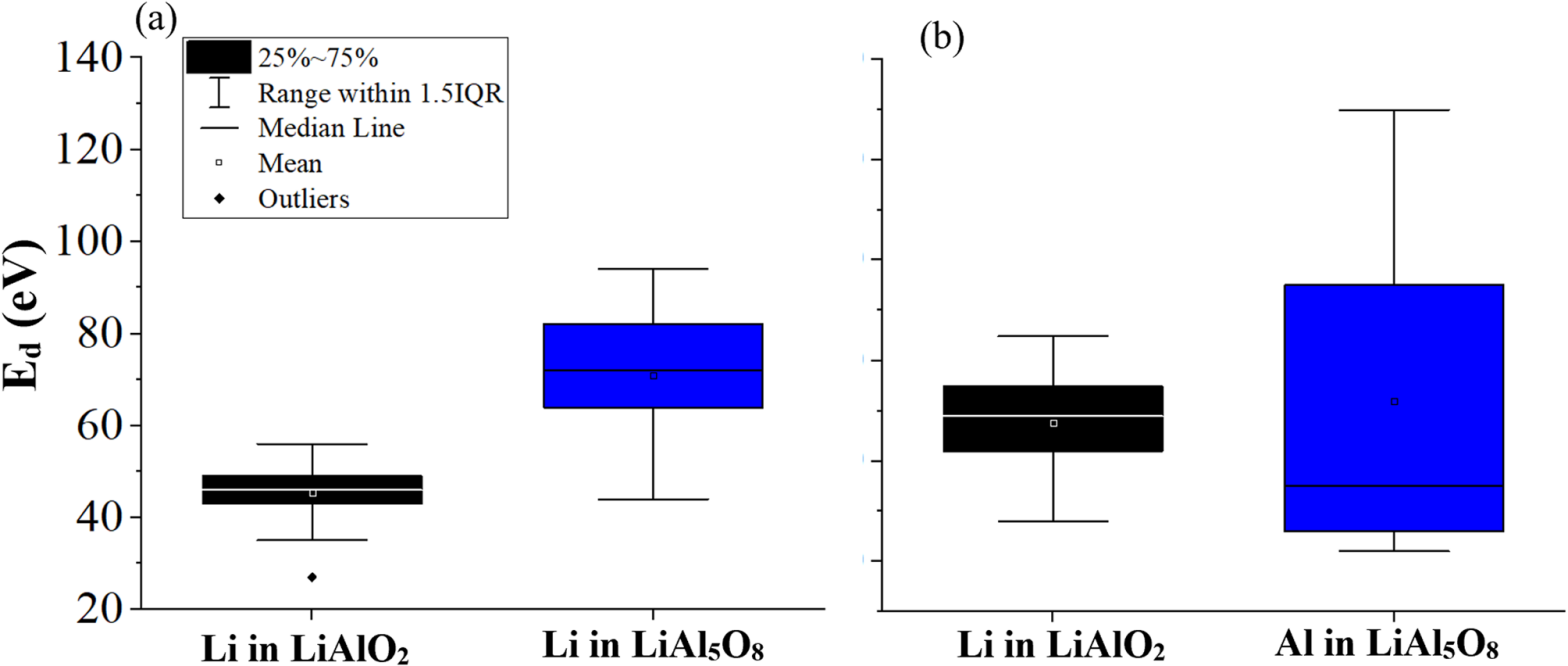
Box and whisker plots of TDE values of (**a**) Li in LiAlO_2_ and LiAl_5_O_8_, (**b**) Al in LiAlO2 and LiAl_5_O_8_.

Calculation of thermal conductivity of pristine γ-LiAlO_2_ was done using reactive force fields (ReaxFF) developed by Shin^40^. The potential was validated by comparing lattice constants, bond lengths, defect formation energies and threshold displacement energies as shown in Tables 1 and 2. The Shin ReaxFF potential with the heat addition/subtraction approach produced thermal conductivity values of ~ 9.91 W/m K for γ-LiAlO_2_ with simulation cell length 300 Å, reasonably close to the experimental value of ~ 13.5 W/m K^41^. This combination was thus used for all subsequent calculations involving defects, temperature dependence, and radiation damage (Fig. 1).

### Thermal conductivity calculation

**Fig. 2:**
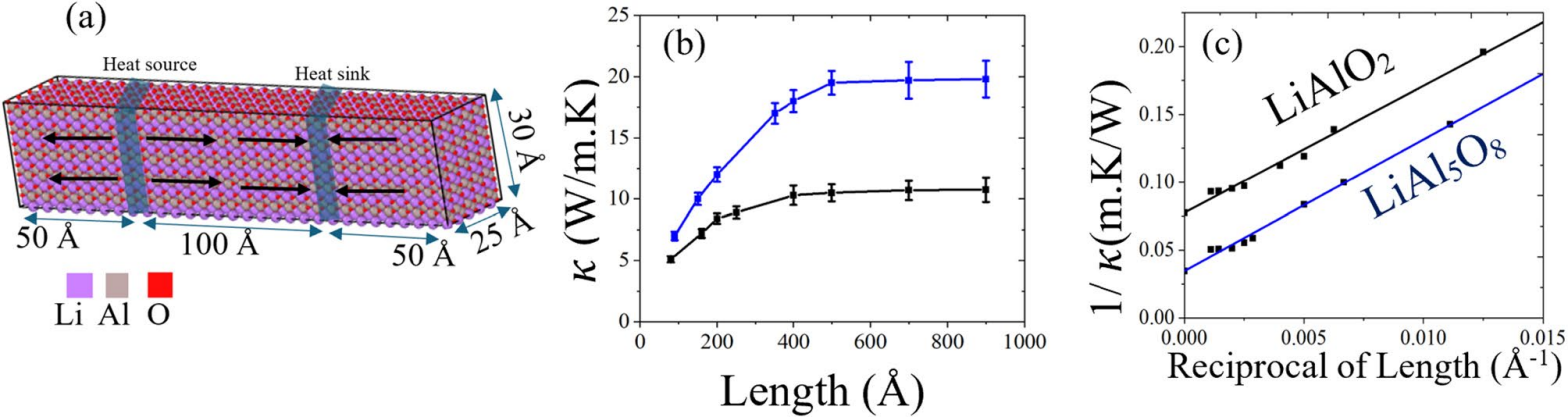
(**a**) Schematic of the γ-LiAlO_2_ supercell illustrating the direct method for thermal conductivity calculations, with heat added and removed in the source and sink regions. (**b**) varIation of κ with system length showing saturation at ~ 400 Å. (**c**) Plot of inverse thermal conductivity (1/κ) versus inverse system length (1/L) for γ-LiAlO_2_ and LiAl_5_O_8_. Extrapolation to infinite length provides bulk thermal conductivity values.

The direct heat addition/subtraction method was applied by dividing the simulation cell into 120 slabs along the transport direction as shown in Fig. 2a. An energy of 0.06 eV was added and removed every timestep to generate a stable thermal gradient. The system was evolved in the microcanonical (NVE) ensemble while maintaining charge equilibration. Temperature profiles were recorded from the 40th and 80th slab after steady state was reached after 100 ps, and thermal conductivity was extracted using Fourier’s law:

$$\:\kappa\:=\frac{J}{{\Delta\:}T/L}$$

where J is the imposed heat flux, ∇T is the linear temperature gradient between the hot and cold regions and L is the distance between the hot and cold regions. All calculations are for single crystal systems that do not include the effect of grain boundaries in a polycrystalline system. To calculate variations in $\:\kappa\:$, five different magnitudes of energy addition and subtraction were used from 0.06 eV-0.10 eV per timestep. For each given simulation cell size, $\:\kappa\:$ values from five different energy addition and subtraction were calculated to obtain the average $\:\kappa\:$ value with standard deviations error bars.

### Radiation damage via primary knock-on atom (PKA) simulations

To simulate irradiation-induced damage, 40 keV primary knock-on atom (PKA) events were introduced into the γ-LiAlO_2_ and LiAl_5_O_8_ supercells of size 15 nm $\:\times\:$ 15 nm $\:\times\:$ 30 nm with ~ 500,000 atoms. In each simulation, a randomly selected Li atom was given an initial velocity corresponding to the target recoil energy, initiating a displacement cascade. After each event, the system was allowed to evolve for 40 ps under NVE conditions to capture cascade evolution and allow short-term dynamic annealing. This process was repeated up to 10 times in the same supercell, resulting in cumulative damage with increasing Frenkel pair concentration. After each PKA event and relaxation period, the thermal conductivity of the damaged structure was recalculated. This method realistically mimics the physics of irradiation but it is limited in achieving high defect densities due to recombination and the finite size of the simulation box. The PKA energies are limited to 40 keV which are constrained by the stability of the interatomic potential. Across all simulations, the highest damage levels reached approximately 200–300 surviving Frenkel pairs, beyond which further accumulation was limited by defect annihilation, cell size and PKA energy. Threshold displacement energies were calculated in 15 directions using same methods used in our previous work to validate the potentials^4^.

### Frenkel pair accumulation

To simulate radiation damage in LiAlO_2_ systems by the formation of Frenkel defect pairs, we employed a Python-based algorithm to selectively displace atoms from their equilibrium lattice sites to interstitial positions. This approach mimics ballistic collision effects during irradiation. The LAMMPS structure data file is parsed to extract both the simulation box dimensions and atom-specific information including ID, atom type (Li, Al, O, or H), and Cartesian coordinates. Each atom is represented in a structured NumPy array to enable vectorized operations. Atoms to be displaced are selected based on user-defined input counts for each atomic species. The critical step is identifying valid interstitial sites for the displaced atoms. Candidate interstitial positions are generated randomly within the simulation box, and each trial position is checked to ensure it lies at least a threshold distance (typically ≥ 5 Å) away from any existing atom. To accelerate this proximity check, the simulation box is divided into a coarse spatial grid, and only atoms in neighboring cells are considered. If a valid interstitial site is found such that the displacement vector exceeds a user-defined minimum length (e.g., 5 Å), the atom’s coordinates are updated to the new position. This method provides a controlled way to create large number of interstitial defects. Details of the method are given in the supplementary information in the form of a flowchart F1.

This approach circumvents the limitations of conventional PKA cascade simulations, which are limited in the number of defects they can produce. Further increasing PKA energy often introduces unstable behavior due to limitations in the interatomic potentials. In contrast, our method allows us to introduce over 100,000 Frenkel defects.

### Effect of vacancy defects 

To isolate the effects of point defects on thermal conductivity, we introduced vacancies by removing 2%, 5%, and 10% of Li or Al atoms from the lattice. A suitable number of oxygen atoms were deleted as well to maintain charge neutrality. The systems were relaxed after deletion to allow local structural equilibration before thermal conductivity calculations. These vacancy concentrations are representative of defect levels arising from prolonged neutron exposure or phase transformation. Thermal conductivity was computed at multiple temperatures (300–900 K) to evaluate how point defect scattering combines with thermal phonon effects. Comparing γ-LiAlO_2_ and LiAl_5_O_8_ under identical defect concentrations allowed for quantitative assessment of their relative radiation tolerance in terms of thermal transport.

### Phonon density of States (PDOS) calculations

To investigate the microscopic mechanisms underlying thermal transport in γ-LiAlO_2_ and LiAl_5_O_8_, we computed the phonon density of states (PDOS). In crystalline materials, heat is predominantly carried by low-frequency acoustic phonons. Therefore, analyzing the PDOS provides valuable insight into how structural disorder and defects influence thermal conductivity at the atomic level.

The PDOS was calculated via the Fourier transform of the velocity autocorrelation function (VACF), which captures vibrational characteristics of the system based on the time evolution of atomic velocities. The VACF is defined as:

$$\:{C}_{i}\left(t\right)=\frac{\langle {v}_{i}\left(0\right).{v}_{i}\left(t\right)\rangle }{\langle {v}_{i}\left(0\right).{v}_{i}\left(0\right)\rangle }$$

where *v*_*i*_*(t)* is the velocity vector of atom *i* at time *t*, and angle brackets denote averaging over time origins and equivalent atoms (e.g., all Li, Al, or O atoms). The normalization ensures that the autocorrelation starts at unity and decays with time as atoms lose memory of their initial vibrational state. The partial PDOS for each atomic species is then computed by applying a cosine Fourier transform to the VACF^42^:

$$\:{g}_{i}\left(w\right)={\int\:}_{0}^{\infty\:}{C}_{i}\left(t\right)\text{cos}\left(\omega\:t\right)dt$$

Here, g_i_ (ω) is the vibrational spectrum (partial PDOS) for atom type *i* as a function of angular frequency ω. By comparing the PDOS of pristine crystals to those with increasing levels of Frenkel pairs or vacancy defects, we directly quantify suppression acoustic modes. These changes correlate strongly with the degradation of thermal conductivity, confirming the role of phonon scattering from lattice disorder.

## Results

The computed densities were 2.51 gm/cc for LiAlO_2_ and 3.53 gm/cc for LiAl_5_O_8_ which agree well with the DFT values of 2.53 gm/cc^43^ and 3.52 gm/cc^43^ respectively thus providing a preliminary validation of the ReaxFF potentials. The bond distances and lattice parameters derived from these potentials are detailed in Table 1 for LiAlO_2_ and Table 2 for LiAl_5_O_8_. For LiAlO_2_ the results show fair agreement with those obtained through DFT^44^ and experimental data^45^. Similarly, the results for LiAl_5_O_8_ are consistent with the DFT data from Materials Project (M.P.)^43^ further validating the suitability of the potentials for both γ-LiAlO_2_ and LiAl_5_O_8_. Defect formation energies for both ceramics were calculated using the same methods as followed in our prior work^6^ and were compared to DFT obtained values. The ReaxFF potential in MD shows a slightly increased vacancy formation energy for all species. Such deviations may be because ReaxFF potentials are parameterized to reproduce a broad range of chemical environments using analytical functions. However, it lacks the explicit treatment of electronic structure and charge density redistribution that DFT includes.

**Table 1:** Calculated bond distances from optimized systems of γ-LiAlO_2_ using ReaxFF compared with experimental results^45^.

Lattice parameters	Calc. (Å)	Exp. (Å)
a	5.42	5.17
c	6.45	6.27

Bond distances AlO_4_	Calc.	Exp.
Al-O (2)	1.84 ± 0.02	1.76
Al-O (2)	1.88 ± 0.02	1.77
O-O (2)	2.99 ± 0.02	2.92
O-O (2)	2.94 ± 0.02	2.89
O-O	2.92 ± 0.02	2.87
O-O	2.81 ± 0.02	2.74

Bond distances LiO_4_	Calc.	Exp.
Li-O (2)	2.21 ± 0.02	2.06
Li-O (2)	1.93 ± 0.02	1.95
O-O (2)	3.31 ± 0.02	3.30
O-O (2)	3.33 ± 0.02	3.29
O-O	3.49 ± 0.02	3.43
O-O	2.82 ± 0.02	2.74

Bond distances (cations)	Calc.	Exp.
Al-Al	3.21 ± 0.02	3.12
Li-Li	3.14 ± 0.02	3.09
Al-Li	2.79 ± 0.02	2.66

Defect formation energy	Calc.	DFT^6^
V_Li_	2.95 eV	2.32 eV
V_Al_	5.46 eV	4.64 eV
V_O_	7.52 eV	6.86 eV

**Table 2:** Calculated bond distances from optimized systems of LiAl_5_O_8_ using ReaxFF compared with DFT results^43^.

Lattice parameters	Calc. (Å)	Materials project (MP) (Å)
a	8.04	7.98

Bond distances AlO_6_	Calc.	MP
Al-O (2)	1.92 ± 0.02	1.96
Al-O (2)	1.84 ± 0.02	1.87
Al-O (2)	1.91 ± 0.02	1.93
O-O (6)	2.61 ± 0.02	2.73
O-O (3)	2.56 ± 0.02	2.59
O-O (2)	2.94 ± 0.02	2.80
O-O (1)	2.89 ± 0.02	2.83

Bond distances AlO_4_	Calc.	MP
Al-O (4)	1.89 ± 0.02	1.78
O-O	3.01 ± 0.02	2.95

Bond distances LiO_6_	Calc.	MP
Li-O (6)	2.21 ± 0.02	2.06
O-O (6)	3.11 ± 0.02	3.03
O-O (6)	2.66 ± 0.02	2.80

Bond distances (cations)	Calc.	MP
Al-Al	2.96 ± 0.02	2.86
Li-Li	4.99 ± 0.02	4.89
Al-Li	2.88 ± 0.02	2.79

Defect formation energy	Calc.	DFT^6^
V_Li_	2.86 eV	2.29 eV
V_Al_	5.52 eV	4.54 eV
V_O_	7.95 eV	7.21 eV

A key aspect to be validated before doing displacement cascades simulations is the threshold displacement energy (TDE)^7,46^. Therefore validating the TDE values for the used potential is crucial for getting reliable results from displacement cascades. The results of the TDE calculations are shown in the box and whisker plots in Fig. 1. Li TDE has a median of 46 eV and 71 eV in LiAlO_2_ and LiAl_5_O_8_ respectively, close to the previously obtained value of 40 eV and 68 eV respectively, from Buckingham potentials in our previous work^4^. Similarly, Al TDE has a median of 69 eV and 55 eV in LiAlO_2_ and LiAl_5_O_8_ respectively, in close agreement to the previously obtained value of 70 eV and 57 eV respectively, from Buckingham potentials in our previous work^4^.

The length-dependence of κ is shown in Fig. 2b for up to 900 Å. It is well known that κ increases with increasing L as longer wavelength phonon modes become active that are dominant in thermal transport^47^. Κ saturates after around 400 Å in both materials which is similar to the convergence lengths observed in other materials like WSe_2_^47^ and Si^39^. Extrapolating κ as a function of inverse system length (κ⁻¹ ∝ L⁻¹), as shown in Fig. 2c, gives a close agreement with experiments. The extrapolated κ values of 12.98 W/m K for LiAlO_2_ approach the reported bulk experimental value of ~ 13.5 W/m K^41^ validating the potential for κ calculation. For the LiAl_5_O_8_ the experimentally reported values for a polycrystal is ~ 24 W/m K for LiAl_5_O_8_^41^. The value obtained from our simulations via the extrapolation method for single crystal is 28.9 W/m K which is in agreement with theoretical expectations that for a single crystal κ is expected to be higher than the experimental polycrystal due to phonon scattering at grain boundaries^41^.

**Fig. 3:**
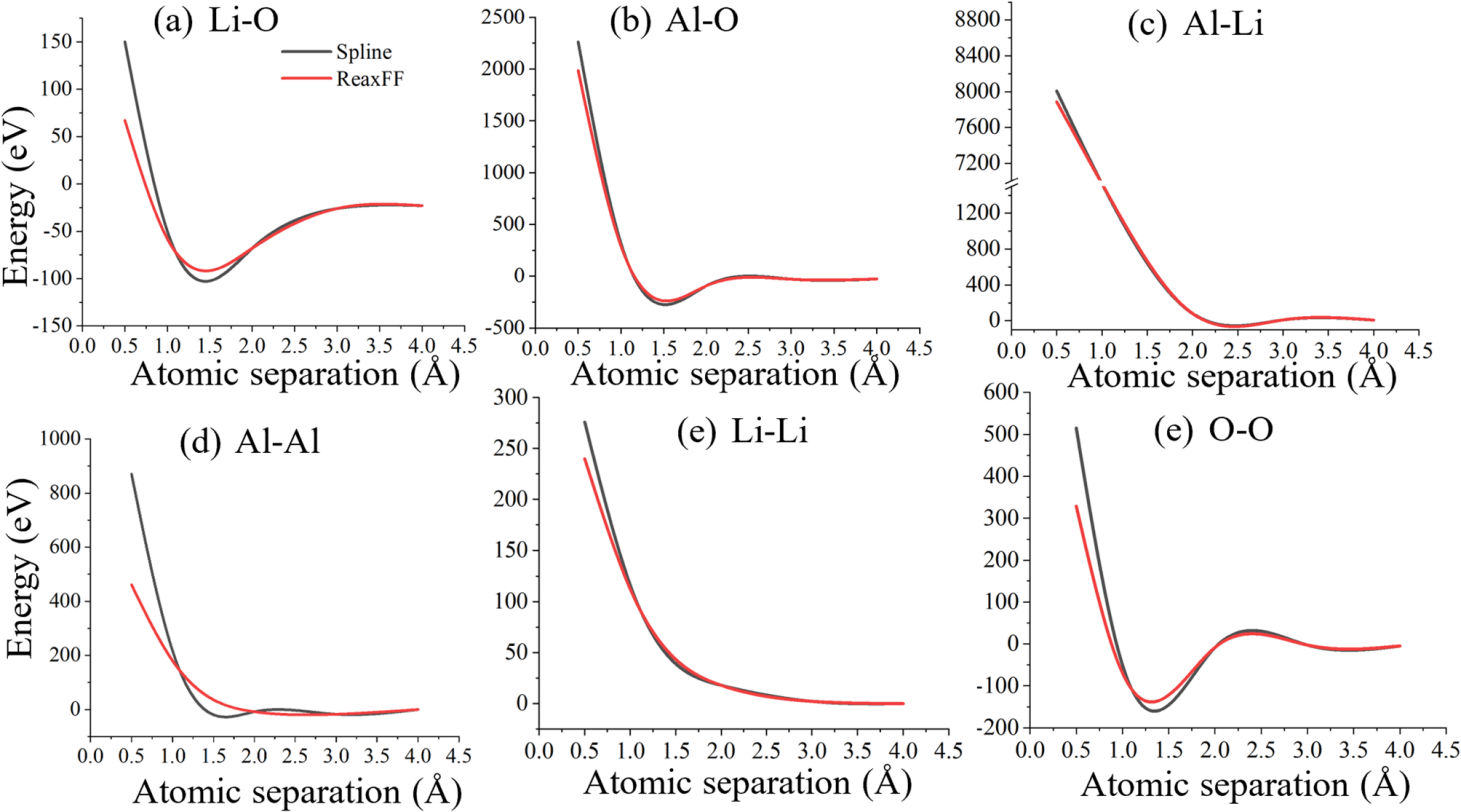
Splined energy versus interatomic distance curves for all relevant atomic pairs after combining the ReaxFF potentials with the Ziegler–Biersack–Littmark (ZBL) short-range repulsion. The plots show the transition from ReaxFF to ZBL repulsion at short distances (< 1 Å) for (**a**) Li–O, (**b**) Al–O, (**c**) Al–Li, (**d**) Al–Al, (**e**) Li–Li, and (**f**) O–O interactions.

Before simulating displacement cascades, the original ReaxFF potentials were splined with the Ziegler–Biersack–Littmark (ZBL) universal repulsive potential to accurately represent short-range interatomic interactions during high-energy collisions. Figure 3a–f show the splined force curves for Li–O, Al–O, Al–Li, Li–Li, Al–Al, and O–O interactions. As expected, these curves demonstrate steep repulsive behavior at interatomic distances below 1 Å, with repulsion energies ranging from several hundred eV to thousands of eV depending on the atomic pair. The steeper repulsion observed in Al–O and Al–Li interactions shows the stronger Coulombic and metallic bonding character of these species. The ZBL-splined potentials ensure realistic modeling of high-energy collision events, where atoms can momentarily approach < 1 Å distances.

**Fig. 4:**
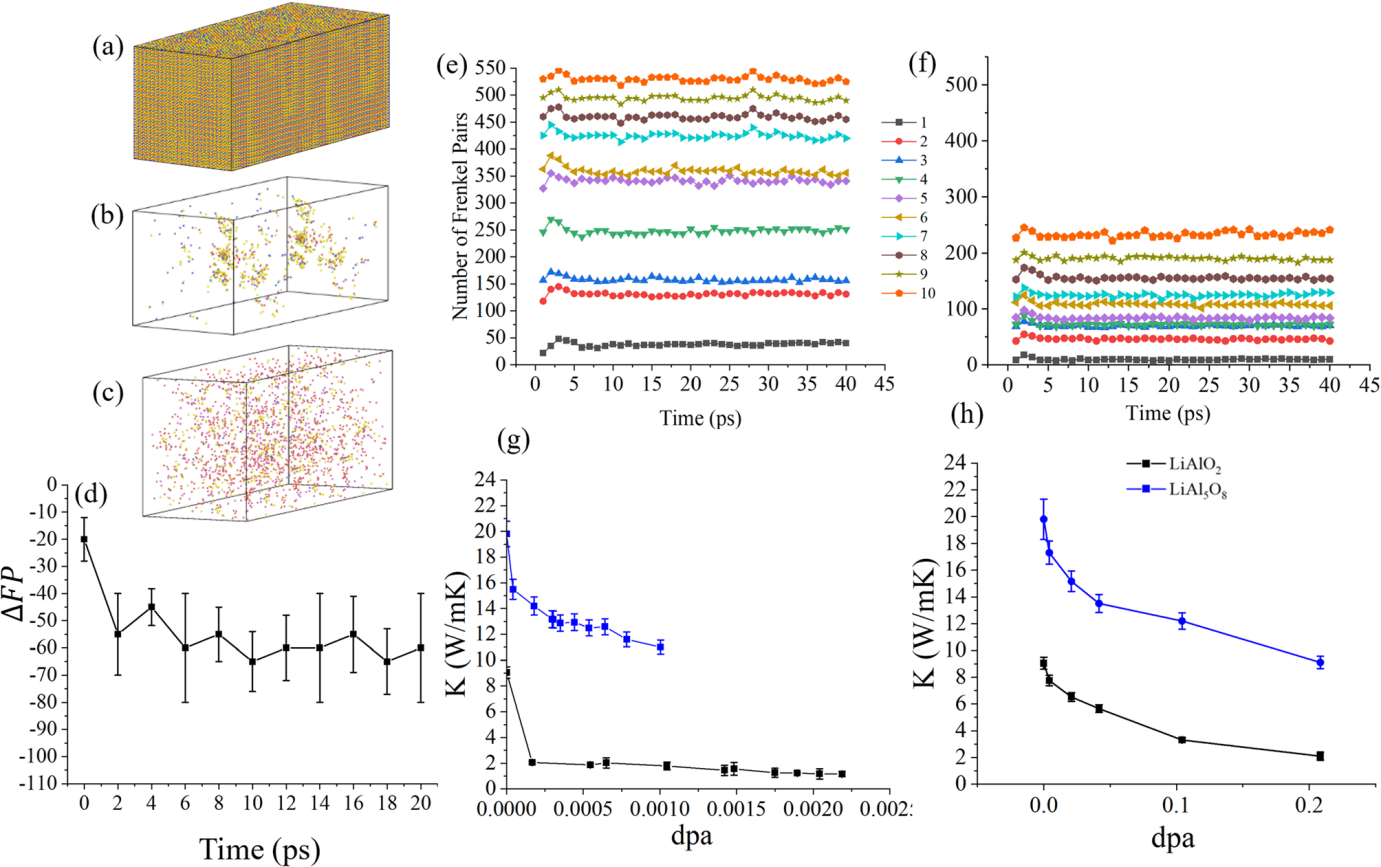
Investigation of radiation-induced thermal transport degradation in LiAlO_2_ and LiAl_5_O_8_. (**a**) The simulation setup consists of a 15 nm × 15 nm × 35 nm supercell containing ~ 500,000 atoms, into which a 40 keV PKA is introduced iteratively 10 times to simulate displacement cascades. (**b**) Formation of a vacancy cluster in LiAlO_2_ following the second PKA event, illustrating local defect agglomeration. (**c**) Uniform defect distribution resulting from the damage accumulation carried out by Python code. (**d**) Defect annealing post 20 ps relaxation in simulation cells produced by damage accumulation algorithm in python. (**e**,**f**) Temporal evolution of cumulative Frenkel pair counts during successive PKA events for (**e**) LiAlO_2_ and (**f**) LiAl_5_O_8_. (**g**) κ degradation as a function of displacement per atom (dpa), computed following each cascade event. (**h**) Comparison of κ reduction resulting from defect accumulation via artificial Frenkel pair insertion, confirming that LiAlO_2_ is more susceptible to phonon scattering from point defects compared to the more structurally resilient LiAl_5_O_8_.

After modifying the interatomic potentials, displacement cascade simulations were initiated in supercells of γ-LiAlO_2_ and LiAl_5_O_8_, each comprising ~ 500,000 atoms, as schematically illustrated in Fig. 4a. In each simulation, a Li atom was randomly selected and imparted with 40 keV of kinetic energy to mimic the effect of a high-energy recoil event, repeated over 10 independent PKA iterations to statistically capture cascade variability. The PKA trajectory spans the full length of the supercell, indicating extensive spatial propagation of the knock-on event. This long-range traversal is characteristic of light ions like Li and it results in a diffuse damage distribution that is not confined to localized volumes. A representative damaged configuration after the second PKA event is visualized in Fig. 4b. The time evolution of damage, quantified by the number of surviving Frenkel pairs (i.e., vacancy-interstitial pairs that do not recombine within the 40 ps simulation window), is shown in Fig. 4e, f for LiAlO_2_ and LiAl_5_O_8_, respectively. LiAlO_2_ accumulates up to ~ 550 Frenkel pairs per cascade, while LiAl_5_O_8_ stabilizes at a significantly lower damage ceiling of ~ 250. This marked contrast corroborates previous findings by Roy et al.^5,8^, which demonstrated that LiAl_5_O_8_ possesses higher TDEs, especially for Al atoms in the spinel framework. The network of corner- and edge-sharing polyhedra in the spinel structure enhances structural rigidity suppressing defect formation.

The thermal conductivity degradation resulting from successive radiation damage is plotted in Fig. 4g for LiAlO_2_ (black line) and LiAl_5_O_8_ (blue line), as a function of displacement per atom (dpa). In γ-LiAlO_2_, the thermal conductivity (κ) plummets from 9.04 W/m K to 2.05 W/m K immediately after the first cascade, reflecting a sharp increase in phonon scattering^48,49^ due to newly formed point defects and small clusters. With subsequent cascades, κ further decreases and saturates near ~ 1.0 W/m K. In contrast, LiAl_5_O_8_ in Fig. 4g exhibits a more gradual degradation, with κ reducing from 19.8 W/m K to 15.5 W/m K after the first event and holding a minimum near 11.1 W/m K after multiple cascades.

This divergence in κ response illustrates the intrinsic radiation tolerance of LiAl_5_O_8_, which stems from its topologically constrained lattice that resists extended defect formation^4^. The higher atomic packing density and mixed ionic-covalent bonding nature in the spinel phase^50^ limit the phonon-defect scattering cross-section by reducing the formation of nanoscale voids and disordered regions. In contrast, the γ-phase of LiAlO_2_, being less densely packed and structurally less rigid, facilitates defect clustering and higher phonon localization, leading to a more pronounced collapse of heat transport pathways.

To probe the effect of higher radiation damage levels beyond the regime accessible by individual displacement cascades, controlled insertion of Frenkel pairs was performed using a custom Python algorithm, as detailed in the Methods section. This approach enabled the simulation of damage levels up to 0.2 dpa, approximately an order of magnitude higher than what was achievable through direct PKA cascades alone. The relaxation of these damage accumulated simulation cells for 20 ps annealed only about 60 Frenkel pairs as shown in Fig. 4d, that are insignificant compared to the large number of Frenkel pairs (10,000-100,000) inserted by python algorithm. So it is safe to assume that more than 99.9% of the inserted defects are surviving during the κ calculation. Interestingly, despite the significantly elevated damage density, the reduction in thermal conductivity was less severe than anticipated. As shown in Fig. 4h, for γ-LiAlO_2_, κ decreased from 9.04 to 2.1 W/m K (77% drop), while in LiAl_5_O_8_, it dropped from 19.8 to 9.1 W/m K (54% drop). These reductions are modest compared to those observed in cascade simulations, where comparable κ degradation occurred at much lower dpa levels. The drop in the thermal conductivity of LiAlO_2_ is comparable to that noticed by Ortiz et al.^41^ for a polycrystalline LiAlO_2_ sample where thermal conductivity dropped from 7.5 W/mK to 1.1 W/mK when irradiated with 120 keV He^+^ ions and 80 keV $\:{D}_{2}^{+}$ ions with fluence of 30 $\:\times\:$ 10^16^ ions/cm^2^.

This anomaly between the two methods can be attributed to the spatial morphology of the introduced defects during the damage accumulation simulations. The inserted Frenkel pairs in damage accumulation simulations through the Python program are uniformly distributed throughout the simulation cell as shown in Fig. 4c, resulting in a homogeneously disordered system devoid of concentrated damage zones. In contrast, displacement cascades inherently produce highly localized damage zones, leading to the formation of defect clusters as shown in Fig. 4b, nanovoids, or even amorphous pockets, which act as efficient phonon scattering centers^51,52^.

**Fig. 5:**
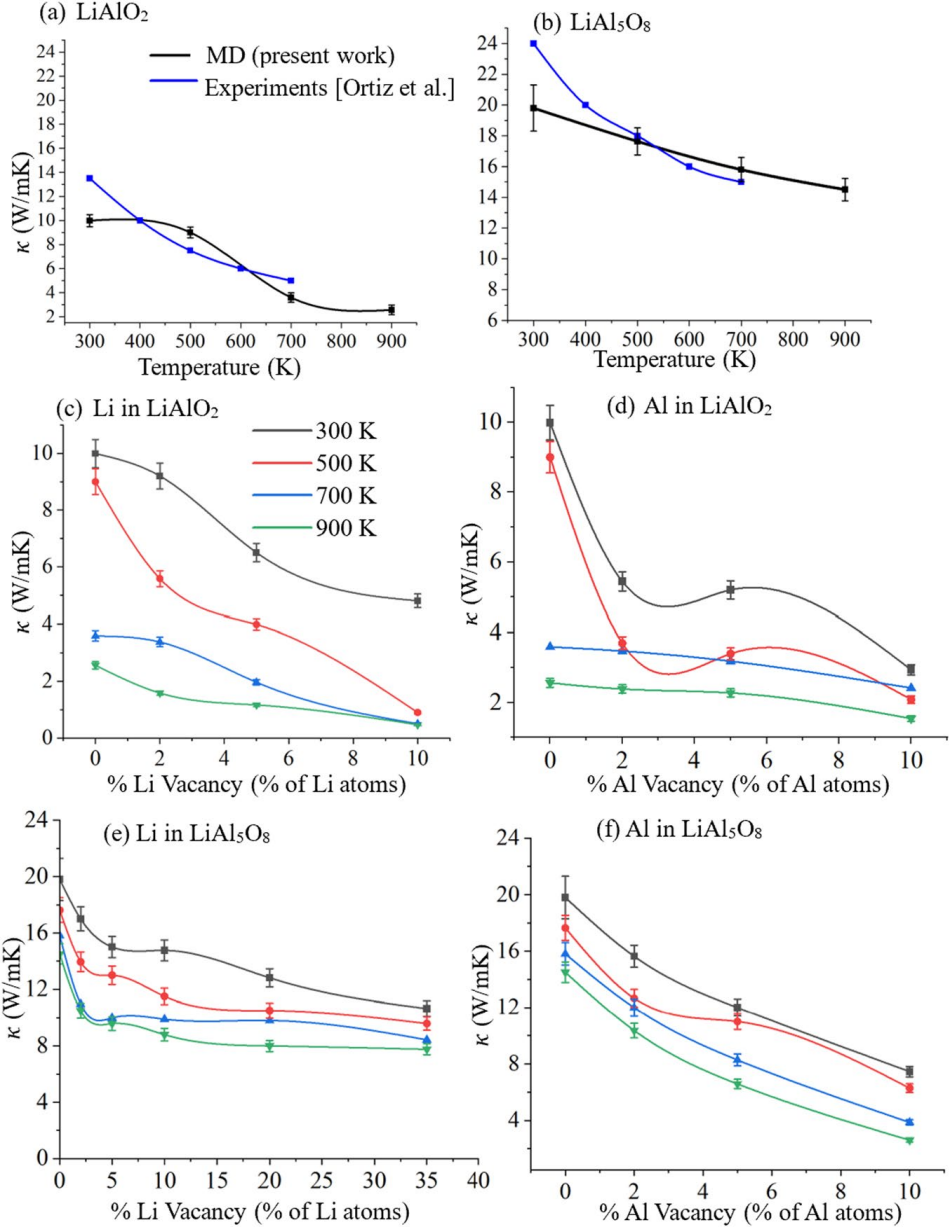
Effect of temperature and species-specific vacancy defects on the thermal conductivity of LiAlO_2_ and LiAl_5_O_8_. Degradation of thermal conductivity as a function of temperature for LiAlO_2_ in (**a**) and for LiAl_5_O_8_ in (**b**) compared with experimental data from^41^. (**c**) Reduction in thermal conductivity of γ-LiAlO_2_ as a function of Li vacancy concentration and (**d**) Al vacancy concentration, which has a more pronounced effect compared to Li vacancies. Thermal conductivity response of LiAl_5_O_8_ to (**e**) Li vacancies, showing relatively modest impact due to the more resilient structure that mitigates phonon scattering effects from isolated Li site disruption. (**f**) Impact of Al vacancies in LiAl_5_O_8_.

The influence of temperature on the degradation of thermal conductivity is plotted in Fig. 5a, b and compared with previous experimental data. For LiAlO_2_, the thermal conductivity decreases from 9.99 W/m K at 300 K to 2.56 W/m K at 900 K. This trend aligns well with experimental observations reported by Ortiz et al.^41^. However, it is important to emphasize that MD simulations inherently represent nanoscale systems, where boundary scattering and finite-size effects dominate. So the absolute values of κ obtained from MD are often lower than experimental bulk measurements. In this context, the temperature dependence, rather than the magnitude, of K is the relevant point of comparison. A similar trend is observed for LiAl_5_O_8_, where κ decreases from 19.8 W/m K at 300 K to 14.5 W/m K at 900 K, matching reasonably with the experimental data.

To investigate the role of intrinsic point defects on heat transport, we simulated the effect of Li and Al vacancies on the thermal conductivity of both γ-LiAlO_2_ and LiAl_5_O_8_ across a range of temperatures. The results are presented in Fig. 5c–f, which detail the vacancy-dependent and temperature-dependent evolution of κ. In γ-LiAlO_2_, Fig. 5c shows that Li vacancies have a pronounced detrimental impact on thermal conductivity. At 300 K, a 10 at% Li vacancy results in a 50% reduction in κ, decreasing from 9.99 W/m K to 4.8 W/m K. At elevated temperatures (T ≥ 500 K), this degradation becomes more severe, with κ plummeting by nearly 82% relative to the defect-free system. The sensitivity of LiAlO_2_ to Li vacancy-induced phonon scattering is amplified by the increased phonon population and anharmonic interactions at higher temperatures^53^, which compound the scattering from mass disorder introduced by vacancies. Even in the absence of Li vacancies, κ naturally decreases with temperature from 9.99 W/m K at 300 K to 2.56 W/m K at 900 K, a ~ 75% reduction, consistent with the expected T⁻¹ behavior of Umklapp scattering in insulating ceramics^54^. The ~ 600 K temperature is relevant for TPBAR operation conditions^1^.

For LiAl_5_O_8_, the absolute lithium content is much lower due to its stoichiometry (7 at% Li vs. 25 at% in LiAlO_2_). To achieve a comparable density of Li vacancies in absolute terms, vacancy concentrations were increased up to 35 at% relative to Li sites. Figure 5e demonstrates that Li vacancies still cause degradation in κ from 19.8 W/m K to 10.6 W/m K at 300 K, representing a ~ 47% drop, slightly lower than the trend observed in LiAlO_2_. However, a key difference emerges in the temperature resilience. For defect-free LiAl_5_O_8_, κ decreases by only ~ 27% over the 300–900 K range, from 19.8 to 14.5 W/m K, compared to the 75% loss in LiAlO_2_. This enhanced thermal stability arises from the structurally rigid and topologically frustrated spinel framework of LiAl_5_O_8_, which likely supports more localized phonon modes and lower anharmonicity in its vibrational spectrum. When considering aluminum vacancies, both materials experience comparable degradation in thermal conductivity. As shown in Fig. 5d, f, increasing Al vacancy content induces up to ~ 70% reduction in κ, reflecting the dominant role of Al–O bonding networks in governing phonon transport in these oxides.

## Discussion

**Fig. 6:**
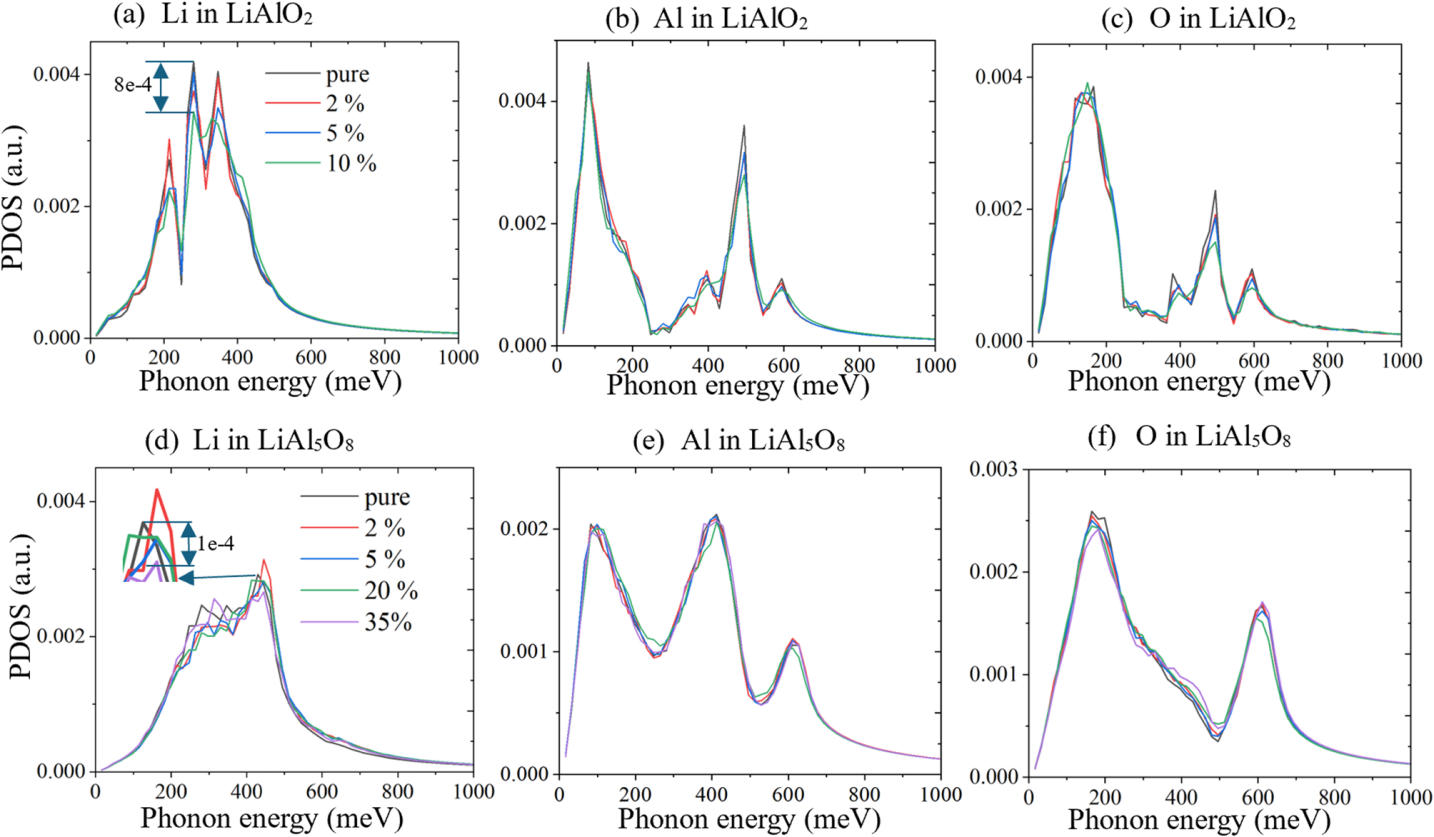
PDOS analysis showing the effect of Li vacancies on vibrational spectra in γ-LiAlO_2_ and LiAl_5_O_8_. (**a**–**c**) PDOS for Li, Al, and O atoms in γ-LiAlO_2_, respectively, comparing the pristine structure with the defected structure containing Li vacancies. The removal of Li causes a suppression in low-frequency vibrational modes, particularly in the Li and Al sublattices, indicating enhanced phonon scattering. (**d**–**f**) Corresponding PDOS for Li, Al, and O atoms in LiAl_5_O_8_. While Li vacancies reduce the vibrational contributions of Li atoms, the effect on Al and O PDOS is less pronounced than in γ-LiAlO_2_, suggesting stronger structural resilience in LiAl_5_O_8_.

The PDOS provides insight into the origin of *κ* degradation under vacancies. Figure 6a–c illustrate the PDOS for Li, Al, and O atoms in LiAlO_2_ for 0%, 2%, 5% and 10% Li vacancy, while Fig. 6d–f portray the same for LiAl_5_O_8_ for 0%, 2%, 5%, 20% and 35% Li vacancy. The PDOS for Li shows significant attenuation in LiAlO_2_, with the peak value reducing by 8$\:\times\:$10^−4^ (arbitrary units). In contrast, the diminished response in LiAl_5_O_8_ is considerably smaller, with peak values reducing only by 1$\:\times\:$10^−4^.

The reduction in PDOS for Li atoms in LiAlO_2_​ is mathematically linked to its observed *κ* degradation due to vacancies. The thermal conductivity can be expressed in terms of the phonon contribution as^55^:

$$\:\kappa\:={\int\:}_{0}^{{\omega\:}_{max}}h\omega\:\langle {v}_{w}^{2}\rangle {\tau\:}_{\omega\:}g\left(\omega\:\right)d\omega\:$$

where $\:\omega\:$ is the phonon frequency, $\:\langle {v}_{w}^{2}\rangle$ is the average squared phonon group velocity, $\:{\tau\:}_{\omega\:}$ is the phonon lifetime, and $\:g\left(\omega\:\right)$ is the PDOS. The introduction of vacancies reduce $\:g\left(\omega\:\right)$, thereby limiting heat conduction channels and reduction in $\:\kappa\:$. In contrast, LiAl_5_O_8_ has a structure that minimizes PDOS suppression, preserving phonon propagation pathways and contributing to its higher *κ* resilience. This disparity in PDOS attenuation stems from differences in the local bonding environments and atomic distribution. Vacancies in LiAlO_2_​ disrupt the phonon spectrum strongly whereas the stoichiometric rigidity and aluminum-rich structure in LiAl_5_O_8_ mitigate these effects, resulting in superior thermal resilience.

## Conclusion

This work investigates the impact of radiation-induced point defects on the thermal transport properties of LiAlO_2_ and LiAl_5_O_8_ ceramics. Under displacement cascades and controlled Frenkel pair insertion, LiAlO_2_ exhibited significantly greater thermal conductivity degradation than LiAl_5_O_8_. For instance, at a damage level of ~ 0.2 dpa, the thermal conductivity of LiAlO_2_ dropped by nearly 77%, whereas that in LiAl_5_O_8_ drops by 54% of its initial conductivity. LiAl_5_O_8_ also showed enhanced resilience to thermal conductivity degradation with increasing temperature, where the conductivity dropped only ~ 27% from 300 K to 900 K, compared to ~ 75% in LiAlO_2_. The percentage decrease in conductivity cannot be directly compared to experimental results, because the current simulations did not consider features found in real ceramics like grain boundaries and pores. However, the trends in thermal conductivity in the two ceramics are of interest to experimentalists. At the atomic level, the PDOS analysis revealed that the introduction of Li and Al vacancies in LiAlO_2_ leads to a pronounced suppression of low- and mid-frequency vibrational modes. The PDOS peak intensity dropped by 8 × 10⁻⁴ in LiAlO_2_ with 10% Li vacancy, compared to a lower 1 × 10⁻⁴ in LiAl_5_O_8_ with 35% Li vacancy concentration. This study reveals that LiAl_5_O_8_ demonstrates superior radiation tolerance and thermal transport under both defect accumulation and high temperature conditions, making it a promising candidate for TPBARs.

## Supplementary Information

Below is the link to the electronic supplementary material.

Supplementary Material 1

## Data Availability

The data and methods reported in this paper are available from the corresponding author upon reasonable request.

## Code availability

The code used for this study is not publicly available, but may be made available on reasonable request from the corresponding author.
