# Supplementary material for: Radiation-induced thermal conductivity degradation in LiAlO2 and LiAl5O8 investigated by molecular dynamics
Source: Sci Rep. 2026 Jan 8;16:1086. doi: 10.1038/s41598-025-26441-y (PMC12783645; doi:10.1038/s41598-025-26441-y)
Supplement: Supplementary file 1 — Supplementary Material 1 [file 41598_2025_26441_MOESM1_ESM.docx]

**SUPPLEMENTARY INFORMATION for**

**Radiation-Induced Thermal Conductivity Degradation in LiAlO₂ and LiAl₅O₈ Investigated by Molecular Dynamics**

Ankit Roy (corresponding author: [ankit.roy@pnnl.gov](mailto:ankit.roy@pnnl.gov)), Andrew M. Casella, Ram Devanathan, Ayoub Soulami and David J. Senor

*^1^Pacific Northwest National Laboratory, Richland, WA 99354, USA*

**Flowchart F1: Algorithm for Generating Frenkel-Type Defect Pairs**

**Start**

**1. Read Input Structure**

- Read LAMMPS charge-style data file.
- Extract box dimensions (xlo/xhi, ylo/yhi, zlo/zhi).
- Parse “Atoms # charge” section and store atom ID, type, charge, and (x, y, z) coordinates.

**2. Select Atoms for Displacement**

- Identify atom indices for Li, Al, and O.
- Randomly choose:
  • n_Li Li atoms
  • m_Al Al atoms
  • k_O O atoms
- Mark remaining atoms as *fixed*.

**3. Generate Interstitial Sites**

- For each selected atom:
  - Randomly propose new position inside box.
  - Check distance to all fixed atoms ≥ min_distance.
  - If valid, compute displacement from original site.
  - Accept only if displacement ≥ min_displacement.
  - Update atom’s position and add to fixed list.

**4. Write Modified Structure**

- Sort atoms by ID.
- Write updated “Atoms # charge” section with new coordinates.
- Save as new LAMMPS data file.

**5. Output Summary**

- Print number of successful displacements and total runtime.
- Output file contains both vacancies and interstitials.

**End**

--------------------------------------------------------------------------------------------------------------------

**ReaxFF reactive force field parameters for LiAlO_2_ and LiAl_5_O_8_ taken from Shin et al. [1]**

Reactive MD-force field: Li/Al/Ti/P/O/H/C

39 ! Number of general parameters

50.0000 !Overcoordination parameter

9.5469 !Overcoordination parameter

1.4254 !Valency angle conjugation parameter

1.7224 !Triple bond stabilisation parameter

6.8702 !Triple bond stabilisation parameter

60.4850 !C2-correction

1.0588 !Undercoordination parameter

4.6000 !Triple bond stabilisation parameter

12.1176 !Undercoordination parameter

13.3056 !Undercoordination parameter

-40.0000 !Triple bond stabilization energy

0.0000 !Lower Taper-radius

10.0000 !Upper Taper-radius

2.8793 !Not used

33.8667 !Valency undercoordination

6.0891 !Valency angle/lone pair parameter

1.0563 !Valency angle

2.0384 !Valency angle parameter

6.1431 !Not used

6.9290 !Double bond/angle parameter

0.3989 !Double bond/angle parameter: overcoord

3.9954 !Double bond/angle parameter: overcoord

-2.4837 !Not used

5.7796 !Torsion/BO parameter

10.0000 !Torsion overcoordination

1.9487 !Torsion overcoordination

-1.2327 !Conjugation 0 (not used)

2.1645 !Conjugation

1.5591 !vdWaals shielding

0.1000 !Cutoff for bond order (*100)

2.0000 !Valency angle conjugation parameter

0.6991 !Overcoordination parameter

50.0000 !Overcoordination parameter

1.8512 !Valency/lone pair parameter

0.5000 !Not used

20.0000 !Not used

5.0000 !Molecular energy (not used)

0.0000 !Molecular energy (not used)

2.0000 !Valency angle conjugation parameter

8 ! Nr of atoms; cov.r; valency;a.m;Rvdw;Evdw;gammaEEM;cov.r2;#

alfa;gammavdW;valency;Eunder;Eover;chiEEM;etaEEM;n.u.

cov r3;Elp;Heat inc.;n.u.;n.u.;n.u.;n.u.

ov/un;val1;n.u.;val3,vval4

C 1.3817 4.0000 12.0000 1.8903 0.1838 0.9000 1.1341 4.0000

9.7559 2.1346 4.0000 34.9350 79.5548 5.9666 7.0000 0.0000

1.2114 0.0000 202.2908 8.9539 34.9289 13.5366 0.8563 0.0000

-2.8983 2.5000 1.0564 4.0000 2.9663 0.0000 0.0000 0.0000

H 0.8930 1.0000 1.0080 1.3550 0.0930 0.8203 -0.1000 1.0000

8.2230 33.2894 1.0000 0.0000 121.1250 3.7248 9.6093 1.0000

-0.1000 0.0000 55.1878 3.0408 2.4197 0.0003 1.0698 0.0000

-19.4571 4.2733 1.0338 1.0000 2.8793 0.0000 0.0000 0.0000

O 1.2450 2.0000 15.9990 2.3890 0.1000 1.0898 1.0548 6.0000

9.7300 13.8449 4.0000 37.5000 116.0768 8.5000 8.3122 2.0000

0.9049 0.4056 68.0152 3.5027 0.7640 0.0021 0.9745 0.0000

-3.5500 2.9000 1.0493 4.0000 2.9225 0.0000 0.0000 0.0000

P 1.5994 3.0000 30.9738 2.3976 0.4904 0.4655 1.3000 5.0000

10.7864 2.7884 5.0000 0.0000 0.0000 3.4186 5.3855 0.0000

-1.0000 3.3786 125.6300 0.5475 11.9674 17.3824 0.0000 0.0000

-13.7379 2.8674 1.0338 5.0000 2.8793 0.0000 0.0000 0.0000

Ti 2.0254 4.0000 47.8800 2.2105 0.1574 0.6311 0.1000 4.0000

12.7041 16.6482 4.0000 0.1000 0.0000 -1.3647 6.8406 0.0000

-1.0000 0.0000 143.1770 27.6505 -0.0753 0.0064 0.8563 0.0000

-15.0000 3.8359 1.0338 12.0000 2.2632 0.0000 0.0000 0.0000

Li 0.0001 1.0000 6.9410 2.6000 0.0865 0.8380 -0.1000 1.0000

9.6984 1.4649 1.0000 0.0000 0.0000 -4.0561 9.7698 0.0000

-1.0000 0.0000 37.5000 5.4409 6.9107 0.1973 0.8563 0.0000

-24.7916 2.2989 1.0338 1.0000 2.8103 1.3000 0.2000 13.0000

Al 2.1967 3.0000 26.9820 2.3738 0.2328 0.4961 -1.6836 3.0000

9.4002 1.6831 3.0000 0.0076 16.5151 -0.3343 6.5000 0.0000

-1.0000 0.0000 78.4675 20.0000 0.2500 0.0000 0.8563 0.0000

-23.1826 1.5000 1.0338 8.0000 2.5791 1.4000 0.2000 13.0000

X -0.1000 2.0000 1.0080 2.0000 0.0000 0.0100 -0.1000 6.0000

10.0000 2.5000 4.0000 0.0000 0.0000 5.0000 9999.9999 0.0000

-0.1000 0.0000 -2.3700 8.7410 13.3640 0.6690 0.9745 0.0000

-11.0000 2.7466 1.0338 2.0000 2.8793 0.0000 0.0000 0.0000

27 ! Nr of bonds; Edis1;LPpen;n.u.;pbe1;pbo5;13corr;pbo6

pbe2;pbo3;pbo4;Etrip;pbo1;pbo2;ovcorr

1 1 158.2004 99.1897 78.0000 -0.7738 -0.4550 1.0000 37.6117 0.4147

0.4590 -0.1000 9.1628 1.0000 -0.0777 6.7268 1.0000 0.0000

1 2 169.4760 0.0000 0.0000 -0.6083 0.0000 1.0000 6.0000 0.7652

5.2290 1.0000 0.0000 1.0000 -0.0500 6.9136 0.0000 0.0000

2 2 153.3934 0.0000 0.0000 -0.4600 0.0000 1.0000 6.0000 0.7300

6.2500 1.0000 0.0000 1.0000 -0.0790 6.0552 0.0000 0.0000

1 3 164.4303 82.6772 60.8077 -0.3739 -0.2351 1.0000 10.5036 1.0000

0.4475 -0.2288 7.0250 1.0000 -0.1363 4.8734 0.0000 0.0000

3 3 142.2858 145.0000 50.8293 0.2506 -0.1000 1.0000 29.7503 0.6051

0.3451 -0.1055 9.0000 1.0000 -0.1225 5.5000 1.0000 0.0000

2 3 160.0000 0.0000 0.0000 -0.5725 0.0000 1.0000 6.0000 0.5626

1.1150 1.0000 0.0000 0.0000 -0.0920 4.2790 0.0000 0.0000

1 4 0.0000 0.0000 0.0000 0.2171 -0.1418 1.0000 13.1260 0.6000

0.3601 -0.2500 20.0000 1.0000 -0.2000 10.0000 1.0000 0.0000

2 4 0.0000 0.0000 0.0000 0.2250 -0.1418 1.0000 13.1260 0.6000

0.3912 -0.1310 0.0000 1.0000 -0.2000 10.0000 0.0000 0.0000

3 4 81.2440 136.3567 0.0000 0.8652 -0.5000 1.0000 25.0000 0.2000

3.5797 -0.2067 16.0316 1.0000 -0.2491 7.9507 1.0000 0.0000

4 4 0.0000 0.0000 0.0000 0.2171 -0.5000 1.0000 35.0000 0.6000

0.5000 -0.5000 20.0000 1.0000 -0.2000 10.0000 1.0000 0.0000

1 5 122.2875 0.0000 0.0000 0.9631 -0.3000 0.0000 36.0000 0.5551

0.3127 -0.2818 16.1571 1.0000 -0.1630 6.8622 0.0000 0.0000

2 5 0.0000 0.0000 0.0000 -0.2872 -0.3000 1.0000 36.0000 0.0082

1.7973 -0.2500 20.0000 1.0000 -0.2578 6.5219 1.0000 0.0000

3 5 130.5629 37.6984 0.0000 0.9228 -0.3000 0.0000 36.0000 0.0850

0.1150 -0.2818 16.1571 1.0000 -0.1343 6.8264 0.0000 0.0000

4 5 0.0000 0.0000 0.0000 -0.2872 -0.3000 1.0000 36.0000 0.0082

1.7973 -0.2500 20.0000 1.0000 -0.2578 6.5219 1.0000 0.0000

5 5 80.1930 0.0000 0.0000 -0.8469 -0.2000 0.0000 16.0000 0.2022

0.7528 -0.1924 14.9725 1.0000 -0.0885 5.0000 0.0000 0.0000

1 6 0.0000 0.0000 0.0000 0.3228 0.3000 0.0000 26.0000 0.6003

1.7161 0.0000 12.0000 1.0000 -0.1015 4.0000 0.0000 0.0000

2 6 0.0000 0.0000 0.0000 1.0000 -0.3000 1.0000 36.0000 0.0100

0.3415 -0.3500 25.0000 1.0000 -0.2770 6.4396 1.0000 0.0000

3 6 65.2411 -0.0200 0.0000 0.1392 0.3000 0.0000 6.0000 0.1482

0.0713 -0.2500 11.9965 1.0000 -0.0713 6.1903 0.0000 0.0000

6 6 0.0000 0.0000 0.0000 0.3228 0.3000 0.0000 26.0000 0.6003

1.7161 0.0000 12.0000 1.0000 -0.1015 4.0000 0.0000 0.0000

5 6 0.0000 0.0000 0.0000 -0.8000 -0.2000 0.0000 16.0000 0.0100

0.5000 0.0000 12.0000 1.0000 -0.1000 8.0000 0.0000 0.0000

2 7 92.8579 0.0000 0.0000 -0.6528 -0.3000 0.0000 36.0000 0.1551

10.0663 -0.3500 25.0000 1.0000 -0.0842 7.1758 0.0000 0.0000

3 7 181.1998 0.0000 0.0000 -0.2276 -0.3000 0.0000 36.0000 0.1925

0.2086 -0.3500 25.0000 1.0000 -0.2000 6.1462 0.0000 0.0000

6 7 0.0000 0.0000 0.0000 1.0000 0.3000 0.0000 26.0000 1.0000

0.5000 0.0000 12.0000 1.0000 -0.2000 10.0000 0.0000 0.0000

7 7 34.0777 0.0000 0.0000 0.4832 -0.3000 0.0000 16.0000 0.5154

6.4631 -0.4197 14.3085 1.0000 -0.1463 6.1608 0.0000 0.0000

4 6 0.0000 0.0000 0.0000 1.0000 0.3000 0.0000 26.0000 1.0000

0.5000 0.0000 12.0000 1.0000 -0.2000 10.0000 0.0000 0.0000

4 7 0.0000 0.0000 0.0000 1.0000 0.3000 0.0000 26.0000 1.0000

0.5000 0.0000 12.0000 1.0000 -0.2000 10.0000 0.0000 0.0000

5 7 0.0000 0.0000 0.0000 0.2500 -0.5000 1.0000 36.0000 0.6000

0.5000 -0.5000 20.0000 1.0000 -0.2000 10.0000 1.0000 0.0000

18 ! Nr of off-diagonal terms; Ediss;Ro;gamma;rsigma;rpi;rpi2

1 2 0.1239 1.4004 9.8467 1.1210 -1.0000 -1.0000

2 3 0.0283 1.2885 10.9190 0.9215 -1.0000 -1.0000

1 3 0.1345 1.8422 9.7725 1.2835 1.1576 1.0637

3 4 0.1472 1.7500 10.1210 1.6250 1.4379 -1.0000

2 5 0.1750 1.7939 13.5000 1.1000 -1.0000 -1.0000

3 5 0.1200 1.8000 10.5000 1.6526 1.4718 -1.0000

1 5 0.2956 1.8399 10.6716 1.6878 -1.0000 -1.0000

2 6 0.4912 1.6088 9.0157 -1.0000 -1.0000 -1.0000

3 6 0.0500 1.8600 9.9536 1.4743 -1.0000 1.0000

5 6 0.3620 2.7000 9.0056 0.0100 -1.0000 -1.0000

2 7 0.0564 1.4937 12.0744 1.7276 -1.0000 -1.0000

3 7 0.2017 1.8458 11.0700 1.6009 -1.0000 -1.0000

6 7 0.3839 1.8924 13.0000 0.0100 -1.0000 1.0000

4 5 0.3928 2.1116 11.2520 0.0100 -1.0000 -1.0000

4 6 0.1939 2.0097 12.9613 0.0100 -1.0000 -1.0000

4 7 0.2000 2.2000 11.3297 0.0100 -1.0000 -1.0000

5 7 0.3918 2.8944 9.0000 0.0100 -1.0000 -1.0000

2 4 0.1744 1.7715 10.4931 0.0100 0.0100 -1.0000

56 ! Nr of angles;at1;at2;at3;Thetao,o;ka;kb;pv1;pv2

1 1 1 59.0573 30.7029 0.7606 0.0000 0.7180 6.2933 1.1244

1 1 2 65.7758 14.5234 6.2481 0.0000 0.5665 0.0000 1.6255

2 1 2 70.2607 25.2202 3.7312 0.0000 0.0050 0.0000 2.7500

1 2 2 0.0000 0.0000 6.0000 0.0000 0.0000 0.0000 1.0400

1 2 1 0.0000 3.4110 7.7350 0.0000 0.0000 0.0000 1.0400

2 2 2 0.0000 27.9213 5.8635 0.0000 0.0000 0.0000 1.0400

1 1 3 53.9517 7.8968 2.6122 0.0000 3.0000 58.6562 1.0338

3 1 3 76.9627 44.2852 2.4177 -25.3063 1.6334 -50.0000 2.7392

2 1 3 65.0000 16.3141 5.2730 0.0000 0.4448 0.0000 1.4077

1 3 1 72.6199 42.5510 0.7205 0.0000 2.9294 0.0000 1.3096

1 3 3 81.9029 32.2258 1.7397 0.0000 0.9888 68.1072 1.7777

3 3 3 80.7324 30.4554 0.9953 0.0000 3.0000 50.0000 1.0783

1 3 2 70.1101 13.1217 4.4734 0.0000 0.8433 0.0000 3.0000

2 3 3 75.6935 50.0000 2.0000 0.0000 1.0000 0.0000 1.1680

2 3 2 85.8000 9.8453 2.2720 0.0000 2.8635 0.0000 1.5800

1 2 3 0.0000 25.0000 3.0000 0.0000 1.0000 0.0000 1.0400

3 2 3 0.0000 15.0000 2.8900 0.0000 0.0000 0.0000 2.8774

2 2 3 0.0000 8.5744 3.0000 0.0000 0.0000 0.0000 1.0421

3 4 3 52.3622 9.6397 1.0379 -12.5000 0.0755 0.0000 3.0000

2 3 4 99.9653 44.2516 0.1000 0.0000 3.0722 0.0000 1.0400

3 3 4 60.0000 40.0000 4.0000 0.0000 1.0000 0.0000 1.0400

3 2 4 0.0000 10.0000 1.0000 0.0000 1.0000 0.0000 1.0400

2 4 3 75.0000 25.0000 2.0000 0.0000 1.0000 0.0000 1.2500

4 3 4 71.2348 36.4753 3.7821 -1.9090 2.5489 0.0000 1.5801

1 3 4 53.2386 27.6683 3.5448 0.0000 0.9129 0.0000 1.2759

3 4 4 70.0000 25.0000 2.0000 0.0000 1.0000 0.0000 1.2500

3 5 3 90.0000 30.4624 2.1468 0.0000 0.0500 0.0000 1.9485

5 3 5 90.0000 5.7486 5.0000 0.0000 2.0000 0.0000 1.1000

3 3 5 62.9344 15.0215 4.3743 0.0000 0.6168 0.0000 1.1673

3 5 5 33.7127 8.0623 3.4580 0.0000 0.0500 0.0000 2.6065

2 3 5 90.0000 9.7766 8.0000 0.0000 0.0505 0.0000 1.7257

1 3 5 61.4655 39.8483 4.2082 0.0000 1.9793 0.0000 1.6005

1 5 1 83.6785 13.3005 2.3764 0.0000 2.0000 0.0000 2.0851

5 1 5 94.4356 19.9912 2.9221 0.0000 1.5337 0.0000 1.6359

1 5 3 66.6190 9.5747 7.8475 0.0000 0.0500 0.0000 1.1000

1 1 5 95.0666 9.4363 4.8061 0.0000 0.4005 0.0000 1.1784

3 6 3 -27.6827 7.7797 2.8604 0.0000 1.3898 0.0000 2.4524

2 3 6 100.0000 0.6912 5.0000 0.0000 0.1035 0.0000 2.0174

6 3 6 91.1462 5.0541 5.7059 0.0000 1.6088 0.0000 1.4410

5 3 6 100.0000 9.1014 3.0000 0.0000 1.3806 0.0000 1.1936

3 2 7 0.0000 4.2750 1.0250 0.0000 1.3750 0.0000 1.4750

2 2 7 0.0000 3.0000 1.0000 0.0000 1.0000 0.0000 1.2500

7 2 7 0.0000 20.2391 0.1328 0.0000 2.9860 0.0000 1.0870

2 3 7 88.1144 13.2143 1.5068 0.0000 3.0000 0.0000 1.0100

3 3 7 34.4326 25.9544 5.1239 0.0000 2.7500 0.0000 1.7141

7 3 7 20.7204 13.4875 4.0000 0.0000 0.6619 0.0000 1.4098

2 7 2 67.4229 4.5148 5.9702 0.0000 3.0000 0.0000 2.6879

2 7 3 41.8108 17.3800 2.6618 0.0000 0.7372 0.0000 1.0100

3 7 3 59.5433 20.0000 4.0000 0.0000 3.0000 0.0000 2.0988

2 7 7 180.0000 -26.7860 7.3549 0.0000 1.0000 0.0000 1.0252

2 7 7 78.2279 37.6504 0.4809 0.0000 1.0000 0.0000 2.9475

6 3 7 39.6976 7.5648 3.1234 0.0000 0.2151 0.0000 1.2915

4 3 5 74.2241 6.7772 2.3445 0.0000 0.6613 0.0000 1.0000

4 3 6 64.9363 10.0731 6.7665 0.0000 1.0000 0.0000 1.9956

4 3 7 88.5948 10.6641 6.5332 0.0000 1.0000 0.0000 1.0000

5 3 7 67.1070 10.0704 3.5000 0.0000 1.1378 0.0000 2.0094

40 ! Nr of torsions;at1;at2;at3;at4;;V1;V2;V3;V2(BO);vconj;n.u;n

1 1 1 1 -0.2500 34.7453 0.0288 -6.3507 -1.6000 0.0000 0.0000

1 1 1 2 -0.2500 29.2131 0.2945 -4.9581 -2.1802 0.0000 0.0000

2 1 1 2 -0.2500 31.2081 0.4539 -4.8923 -2.2677 0.0000 0.0000

1 1 1 3 1.2799 20.7787 -0.5249 -2.5000 -1.0000 0.0000 0.0000

2 1 1 3 1.9159 19.8113 0.7914 -4.6995 -1.0000 0.0000 0.0000

3 1 1 3 -1.4477 16.6853 0.6461 -4.9622 -1.0000 0.0000 0.0000

1 1 3 1 0.4816 19.6316 -0.0057 -2.5000 -1.0000 0.0000 0.0000

1 1 3 2 1.2044 80.0000 -0.3139 -6.1481 -1.0000 0.0000 0.0000

2 1 3 1 -2.5000 31.0191 0.6165 -2.7733 -2.9807 0.0000 0.0000

2 1 3 2 -2.4875 70.8145 0.7582 -4.2274 -3.0000 0.0000 0.0000

1 1 3 3 -0.3566 10.0000 0.0816 -2.6110 -1.9631 0.0000 0.0000

2 1 3 3 -1.4383 80.0000 1.0000 -3.6877 -2.8000 0.0000 0.0000

3 1 3 1 -1.1390 78.0747 -0.0964 -4.5172 -3.0000 0.0000 0.0000

3 1 3 2 -2.5000 70.3345 -1.0000 -5.5315 -3.0000 0.0000 0.0000

3 1 3 3 -2.0234 80.0000 0.1684 -3.1568 -2.6174 0.0000 0.0000

1 3 3 1 1.1637 -17.3637 0.5459 -3.6005 -2.6938 0.0000 0.0000

1 3 3 2 -2.1289 12.8382 1.0000 -5.6657 -2.9759 0.0000 0.0000

2 3 3 2 2.5000 -22.9397 0.6991 -3.3961 -1.0000 0.0000 0.0000

1 3 3 3 2.5000 -25.0000 1.0000 -2.5000 -1.0000 0.0000 0.0000

2 3 3 3 -2.5000 -2.5103 -1.0000 -2.5000 -1.0000 0.0000 0.0000

3 3 3 3 -2.5000 -25.0000 1.0000 -2.5000 -1.0000 0.0000 0.0000

0 1 2 0 0.0000 0.0000 0.0000 0.0000 0.0000 0.0000 0.0000

0 2 2 0 0.0000 0.0000 0.0000 0.0000 0.0000 0.0000 0.0000

0 2 3 0 0.0000 0.1000 0.0200 -2.5415 0.0000 0.0000 0.0000

0 1 1 0 0.0000 50.0000 0.3000 -4.0000 -2.0000 0.0000 0.0000

1 1 3 3 -0.0002 20.1851 0.1601 -9.0000 -2.0000 0.0000 0.0000

1 3 3 1 0.0002 80.0000 -1.5000 -4.4848 -2.0000 0.0000 0.0000

3 1 3 3 -0.1583 20.0000 1.5000 -9.0000 -2.0000 0.0000 0.0000

1 1 1 4 -0.3232 14.3871 0.1823 -9.8682 -1.7255 0.0000 0.0000

4 1 1 4 -0.1452 50.0000 -0.1915 -8.0773 -1.7255 0.0000 0.0000

0 1 4 0 4.0000 45.8264 0.9000 -4.0000 0.0000 0.0000 0.0000

0 4 4 0 4.0000 45.8264 0.9000 -4.0000 0.0000 0.0000 0.0000

4 3 4 3 0.1946 20.0266 -0.3314 -8.1095 0.0000 0.0000 0.0000

2 1 3 4 -0.1220 61.5112 0.3316 -5.4970 0.0000 0.0000 0.0000

2 3 4 3 -1.5000 -1.0000 0.3045 -2.5000 0.0000 0.0000 0.0000

1 3 4 3 -0.9451 8.2456 0.5757 -5.7138 0.0000 0.0000 0.0000

2 1 3 5 0.0000 84.3556 0.1000 -3.1953 0.0000 0.0000 0.0000

1 1 3 5 0.0000 51.0461 0.1059 -7.2043 0.0000 0.0000 0.0000

2 3 5 3 -0.2500 0.0100 -0.5000 -4.6984 0.0000 0.0000 0.0000

2 3 6 3 0.0918 64.5743 -1.0000 -4.4228 0.0000 0.0000 0.0000

1 ! Nr of hydrogen bonds;at1;at2;at3;Rhb;Dehb;vhb1

3 2 3 2.1200 -3.5800 1.4500 19.5000

[1] Y.K. Shin, M.Y. Sengul, A. Jonayat, W. Lee, E.D. Gomez, C.A. Randall, A.C. Van Duin, Development of a ReaxFF reactive force field for lithium ion conducting solid electrolyte Li 1+ x Al x Ti 2− x (PO 4) 3 (LATP), Physical Chemistry Chemical Physics 20(34) (2018) 22134-22147.
